# Seismic performance evaluation of plastered cellular lightweight concrete (CLC) block masonry walls

**DOI:** 10.1038/s41598-023-37159-0

**Published:** 2023-07-04

**Authors:** Khalid Khan, Khan Shahzada, Akhtar Gul, Inayat Ullah Khan, Sayed M. Eldin, Mudassir Iqbal

**Affiliations:** 1 Department of Civil Engineering, University of Engineering and Technology, Peshawar, 200240 Pakistan; 2 Center of Research, Faculty of Engineering, Future University in Egypt, New Cairo, 11835 Egypt

**Keywords:** Civil engineering, Structural materials, Composites

## Abstract

The current research presents a novel and sustainable load-bearing system utilizing cellular lightweight concrete block masonry walls. These blocks, known for their eco-friendly properties and increasing popularity in the construction industry, have been studied extensively for their physical and mechanical characteristics. However, this study aims to expand upon previous research by examining the seismic performance of these walls in a seismically active region, where cellular lightweight concrete block usage is emerging. The study includes the construction and testing of multiple masonry prisms, wallets, and full-scale walls using a quasi-static reverse cyclic loading protocol. The behavior of the walls is analyzed and compared in terms of various parameters such as force–deformation curve, energy dissipation, stiffness degradation, deformation ductility factor, response modification factor, and seismic performance levels, as well as rocking, in-plane sliding, and out-of-plane movement. The results indicate that the use of confining elements significantly improves the lateral load capacity, elastic stiffness, and displacement ductility factor of the confined masonry wall in comparison to an unreinforced masonry wall by 102%, 66.67%, and 5.3%, respectively. Overall, the study concludes that the inclusion of confining elements enhances the seismic performance of the confined masonry wall under lateral loading.

## Introduction

To fulfill the modern-day demand of sustainable and green building construction, several eco-friendly and lightweight masonry materials have been introduced^1^. The importance of sustainable and energy-efficient masonry materials cannot be ignored even in high seismicity regions of the world^2,3^. Among these novel and sustainable materials, foam concrete (FC) is one of the attention triggering lightweight construction materials, which is composed of cement, sand, fly ash, water, and foaming agents^4–6^. Other academic names used for FC are cellular lightweight concrete (CLC)^7^ and low-density concrete^8^. FC is an innovative type of material having low density due to the incorporation of foaming agents, which introduce micro air bubbles^9^. FC can be lighter up to 87% as compared to conventional concrete and its density varies from 300 to 1840 kg/m^3^^10–13^. On the other hand, autoclaved aerated concrete (AAC) which is also a type of lightweight concrete consists of cement, fuel ash, sand, lime, aluminum powder and water^14^.

A number of countries have started using FC in the building construction sector owing to the low thermal conduction, lightweight, economical, and eco-friendly perspectives^15–17^. Thermal conductance of FC is considered as 5–30% that of conventional concrete and ranges from 0.1 to 0.7 W/mk^16,18^. The thickness of a conventional concrete wall would be five times more than FC to achieve the same value of thermal conductivity^19^. Slightly increasing the thickness of the wall, will eliminate the need for thermal insulations layers in the weather condition of northern areas of Pakistan. As almost 60–70% of total operational energy is lost through walls, roofs, and other enveloping structures, and people of northern areas of Pakistan pay 13th time their earnings on fuels for heating their houses^20^. The energy required for cooling and heating of building depends on the properties of construction materials^21^. Therefore, improvement of the envelope structures are needed to conserve building energy^22^. FC has good fire resistance, as Camille Laurent, 2014 performed an experimental investigation on FC fire resistance under insulation criteria up to 900 °C and found that FC has better performance as compared to conventional concrete^23^. Other research studies illustrate that the strength degradation of low-density FC during fire is very low as compared to conventional concrete^6,24^.

The FC is used in concrete structures to reduce dead load and consequently reduce the sizes of structural elements. As a result of being lightweight, the seismic forces attracted due to the inertia of the structural elements would reduce^3,25–27^. Zade et al.^28^ evaluated the seismic performance of the CLC infill reinforced concrete (RC) frame structure and found that the CLC infill shows one of the most vulnerable seismic performances compared to other infill materials. It was attributed to the low shear-bond strength of the CLC block infill RC frame. However, the probability of collapse of the CLC block infill RC frame was satisfied and found within limit of design code^28^. Chourasia et al.^29^ evaluated the seismic behavior of CLC panels and concluded that these panels are suitable for constructing of low-to-medium rise buildings in seismic regions^29^. On the other hand, most of the literature is only focused on the mechanical and physical properties of cellular lightweight concrete block masonry (CLCBM)^27,30,31^. However, seismic performance of FC or CLCBM are found to be scared in literature. The compressive strength of such masonry units is very low and can be used in a non-seismic zone limited to a number of stories. Most of the CLC block units have compressive strength lower than required for construction in seismic zone as per the Eurocode 8^32^. Therefore, a proper experimental investigation is needed before recommending such masonry units for construction in high seismic zones and considering them suitable for construction.

Keeping in view the versatile features of CLC in terms of eco-friendly, lightweight, low cost and easy in construction, the CLCBM requires the attention of researchers, to be evaluated as a load bearing walls. Therefore, it is the prime need of the day to assess the performance of CLCBM against lateral loading, with the configuration of load bearing walls, to achieve the aim of this research work two walls, one confined masonry (CM) and other unreinforced masonry (URM) were constructed and tested in Structural Laboratory of University of Engineering and Technology (UET), Peshawar.

## Materials and methods

**Figure 1:**
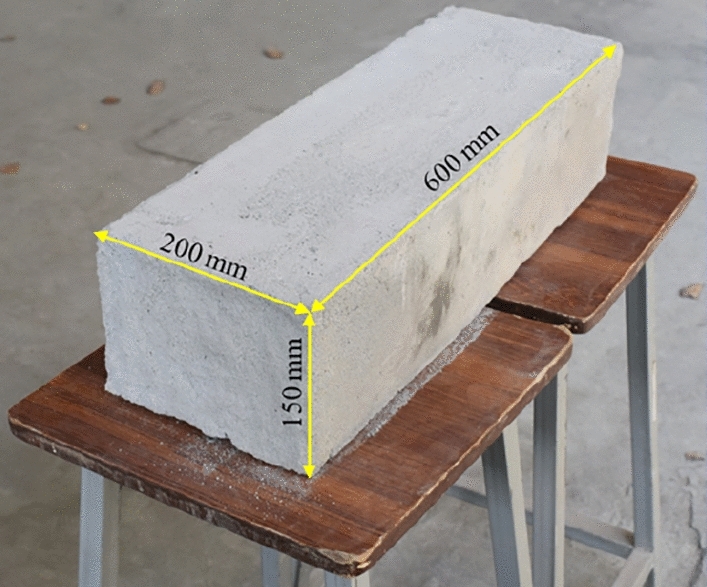
CLC block.

CLC blocks, used in this research study are made of cement, sand, fly ash, water, and foaming agent. The basic constituents such as cement, sand (fine), fly ash, water, and foaming agents were used (per cubic meter of CLC) in amount of 95 kg, 123 kg, 260 kg, 232 kg, and 40 kg, respectively. The dimension of the CLC block is shown in Fig. 1.

The elementary mechanical properties of CLC block and masonry influence the overall seismic performance of the structure and hence are key design parameters. CLC block used in this research study are fabricated with locally available constituent materials. Therefore, to verify the basic strength and to define loading protocols for full scale structure, basic mechanical properties of CLC blocks are assessed.

### Material properties

The mechanical properties were achieved from the testing of blocks, masonry prisms, and masonry wallets. All these tests were performed according to relevant ASTM standards and available literature, listed in Table 1. Tests for CLC blocks contain compressive strength (*f*_*cb*_), flexural strength (*f*_*ctb*_), and thermal conduction (*U*). The compressive strength (*f*_mtm_), tensile strength (*f*_tm_), modulus of elasticity (*E*_mm_), and modulus of rigidity (*G*_mm_) of masonry are also found and listed in Table 1. Three (03) samples were used for each test. The ratio of *G*_*m*_*/E*_*m*_ is 0.36, which is in close agreement with the range (0.1–0.4) recommended by Tomasevic (1999)^33^. However, Eurocode 6^31^ proposes the value of *G*_*m*_*/E*_*m*_ equal to 0.4. It is also worth noting that 0.36 is close to the upper limit of the ratio, which shows that CLC is a weak material.

**Table 1:** Mechanical and physical properties of CLC block and masonry.

Parameters	Description		No. of samples	References	Average values
*f*_cb_	Compressive strength of CLCBs, MPa [CV]		5	^32^	1.38 [1.20%]
*f*_ctb_	Flexure strength of CLCBs, MPa [CV]		5	^33^	0.41 [1.03%]
*U*	Thermal conductivity, W/mk [CV]		5	^34^	0.36 [2.12%]
*f*_mtm_	Compressive strength of CLCBM, MPa [CV]		3	^35^	0.43 [3.00%]
*f*_tm_	Tensile strength of CLCBM, MPa [CV]		3	^36^	0.10 [2.34%]
*E*_mm_	Elastic modulus of CLCBM, MPa [CV]		3	^34^	130.87 [2.11%]
*G*_mm_	Shear modulus of CLCBM, MPa [CV]		3	^34^	47.81 [1.98%]
*G*_*mm*_*/E*_*mm*_	Ratio of shear and elastic modulus		–	–	0.36
*ρ*_*m*_	Density of CLCB, kg/m^3^ [CV]		5	^32^	750 [1.8%]
*f*_*y*_	Yield strength of steel, MPa [CV]		3	^37^	0.42 [1.13%]

### Construction of full-scale walls

The in-plane seismic performance of two full-scale walls including CM and URM walls, was assessed. All specimens were plastered, and masonry units were placed in a mortar of 1:4 (cement:sand) mixture. The concrete used in confining elements and RC foundation was of 1:2:4. The concrete mixture is the ratio of cement, fine aggregate (FA) and coarse aggregate (CA) with cement. This ratio was adopted because of the traditional and mostly used mixture ratio in construction industry in Pakistan. Reinforcements used in confining elements were No.13 (4#) and No.10 (3#). These reinforcements were of grade 60 and corresponding yield strength are given in Table 1. Both walls have dimensions of 3068 mm in width, 3050 mm in height and 220 mm in thickness. A window opening of size 762 mm × 915 mm in length and height was provided in each wall. The overall area of window is less than 10% of the wall gross area, which is in accordance with the Eurocode 6^31^.

**Figure 2:**
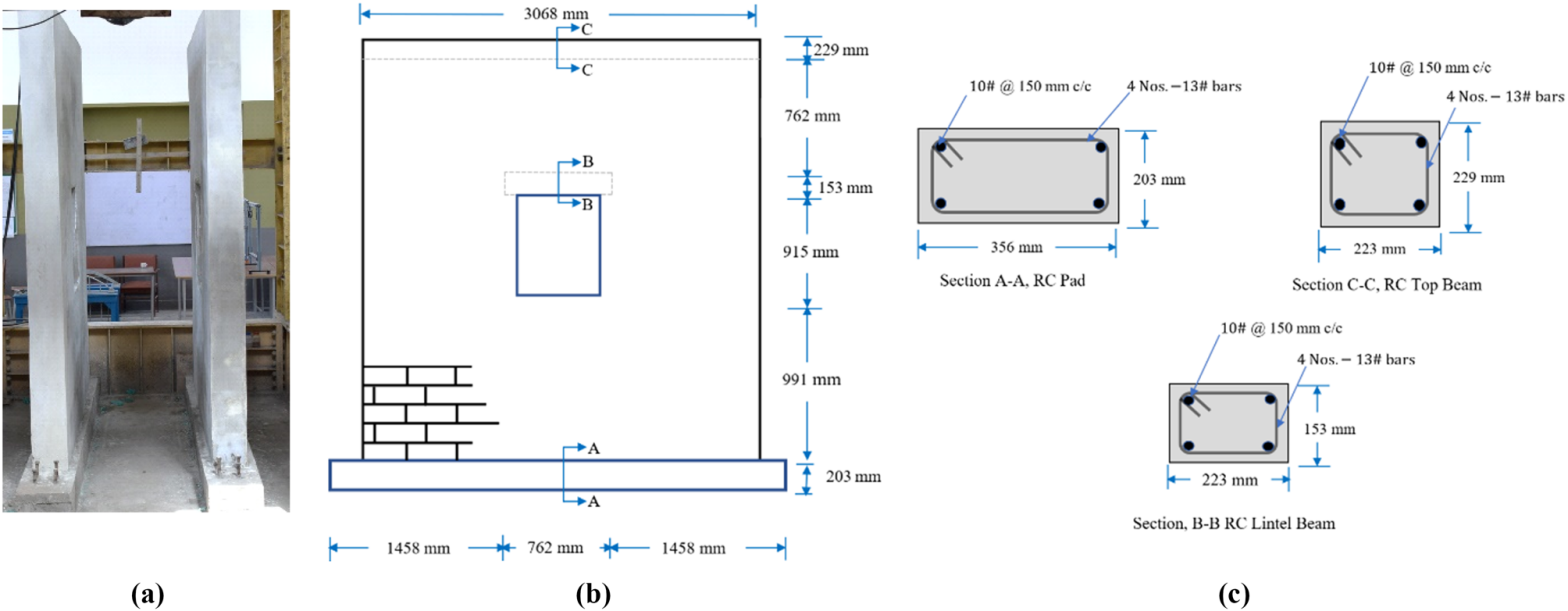
(**a**) Walls ready for testing; (**b**) dimensions of URM wall; (**c**) section details.

In order to simulate actual conditions as exists in building, both walls were laid on a RC foundation of dimension 356 mm in width, 203 mm in height and 3658 mm length, with mortar. The RC foundations were attached to a strong floor through high tension bolts, in order to avoid uplifting and sliding of foundation. For monolithic action the reinforcements of the columns were fixed in the RC foundation. To uniformly distribute the vertical load, tie beam of size 223 mm × 229 mm was provided at the top of both walls. Masonry units were placed in mortar of average thickness of 10 mm in staggered pattern to avoid the connectivity of vertical joints. Plaster was applied on both specimens. The full-scale walls of CLCBM are shown in Fig. 2.

**Figure 3:**
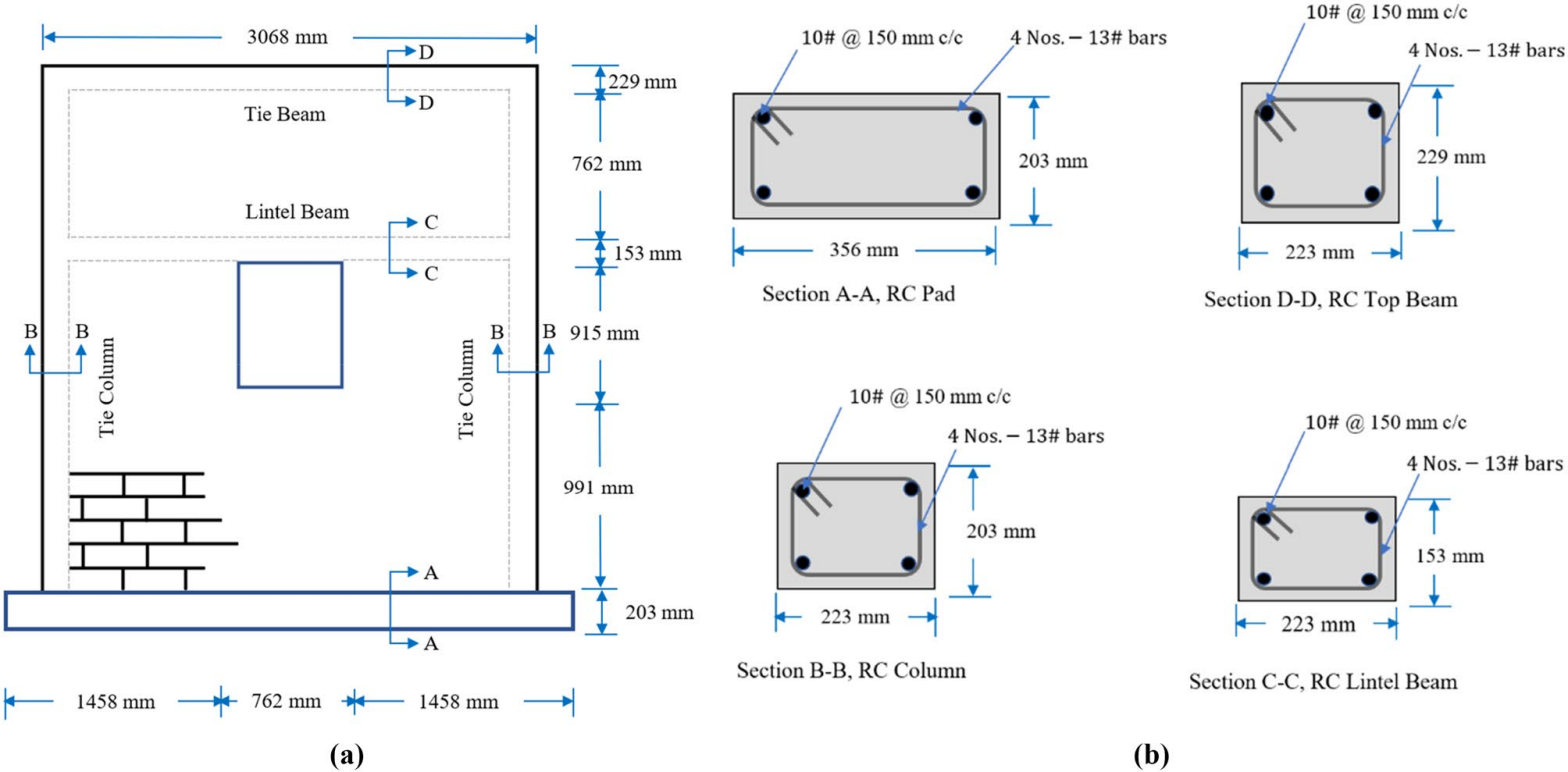
Illustrations of (**a**) CM wall; (**b**) section details.

CM wall consists of tie columns of size 223 mm × 203 mm and one full-length lintel beam of size 223 mm × 153 mm while in case of URM wall, the lintel beam used only provided over the opening. Confining elements were reinforced with four No.13 (4#) as longitudinal bars and transverse reinforcement as No.10 (3#) bars @ 150 mm center to center (c/c). Confining elements have the same dimensions and details for both specimens. The sectional and wall dimension details are listed in Table 2 and as shown in Figs. 2 and 3. The size and reinforcement of confining elements is within range recommended by Tomazevic^33^. To reflect the actual scenario of the field, plaster having thickness of 10 mm and of 1:4 was used for both samples. Mortar cubes of the same ratio were prepared and tested for compressive strength, the average compressive strength of four cubes was 9.34 mpa tooting of length 63.5 mm were provided on the internal face of CM wall to ensure proper connection between masonry and confining element. Both specimens were whitewashed to increase the crack’s visibility.

**Table 2:** Complete detail of specimens.

Wall type	RC pad	Tie columns	Top tie beam	Lintel beam	Opening
CM wall	356 mm × 203 mm	223 mm × 203 mm	223 mm × 229 mm	223 mm × 153 mm	762 mm × 915 mm
Longitudinal reinforcements	4 Nos.13# bars	4 Nos.-13# bars	4 Nos.-13# bars	4 Nos.13# bars	–
Transverse reinforcements	10# @150 mm c/c	10# @150 mm c/c	10# @150 mm c/c	10# @150 mm c/c	–
URM wall	356 mm × 203 mm	–	223 mm × 229 mm	223 mm × 153 mm	762 mm × 915 mm
Longitudinal reinforcements	4 Nos.13# bars	–	4 Nos.-13# bars	4 Nos.13# bars	–
Transverse reinforcements	10# @150 mm c/c	–	10# @150 mm c/c	10# @150 mm c/c	–

A cantilever boundary system (free at the top and fixed at the bottom) was ensured, with a constant pre-compression load (to simulate gravity load) of 60 kN applied through a vertical hydraulic jack of capacity of 500 kN, corresponding to the average compressive stress of σ_o_ = 0.08 MPa for each specimen. The load applied by hydraulic jack is uniformly distributed to wall through steel girder, and steel plates, placed at the top of the wall. Usually, the pre-compression load on masonry structure ranges from 10 to 15%^1^, and 15 to 20%^38^ of the compressive strength of the masonry. In this study, 20% of the compressive strength of the masonry was applied as a pre-compression load. In the present study, the vertical axial load on walls was applied as 0.20*f*_*mtm*_, following the recommendation of Calderini et al.^38^. A thin layer of sand was needed at the top of wall to uniformly distribute the vertical load.

### Instrumentations

**Figure 4:**
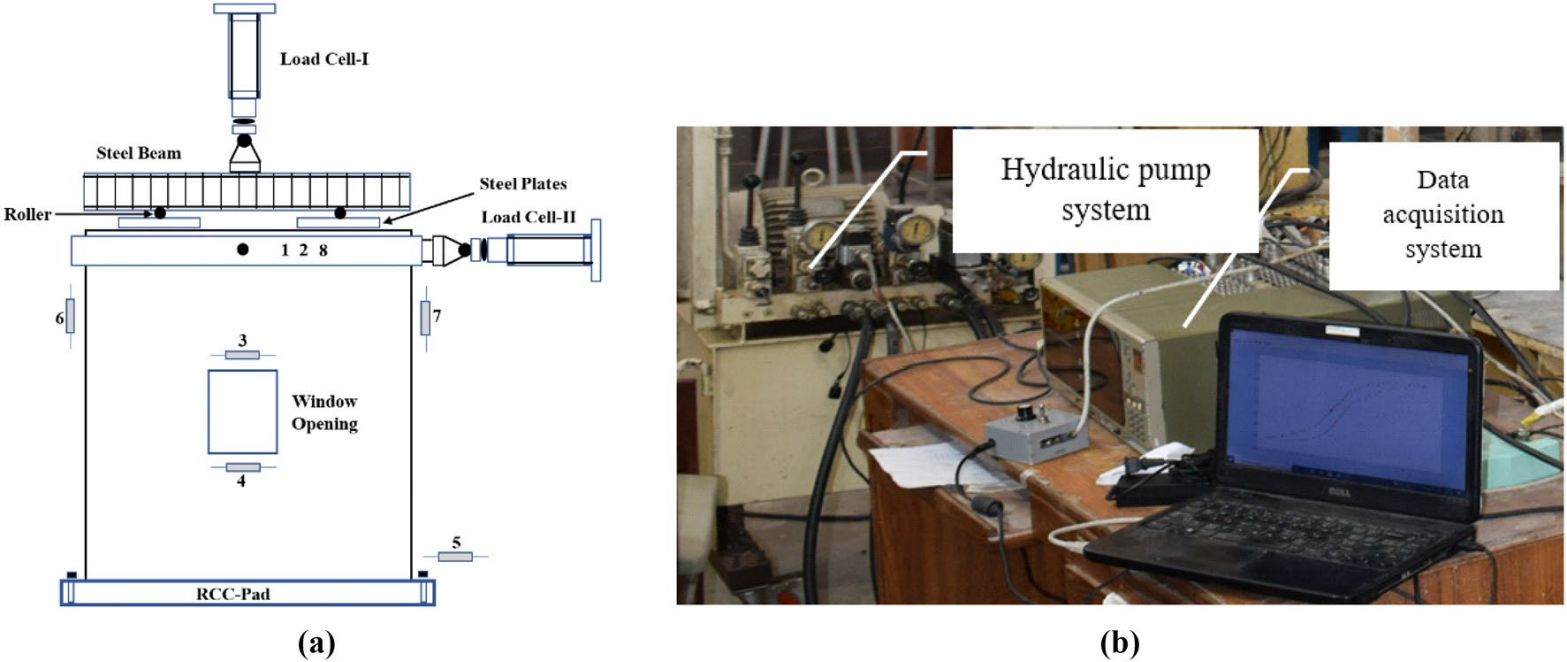
(**a**) Instrumentation of the wall (2D) and (**b**) data acquisition system.

The instrumentation of the walls has been shown in Fig. 4. Two load cells were employed to measure the vertical load and horizontal load, respectively. Eight linear variable displacement transducers (LVDTs) were used to record the displacement field at different locations of the wall. The first two LVDTs 1 and 2 were attached to the center of the top beam and used as control gauges. LVDT-3 was mounted to the center of lintel beam to monitor the relative displacement, while LVDT-4 was used for sill level displacement measurement. LVDT-5 was placed at the bottom of the wall to measure any in-plane sliding. LVDT-6 and LVDT-7 were installed to record the vertical displacement produced due to uplifting of the wall. LVDT-8 was used for measurement of the out-of-plane displacement field. LVDTs 1 and 2 were having displacement measurement capacity of 150 mm while the rest of the LVDTs can measure displacement up to 50 mm. All these LVDTs were connected to the data acquisition system, as shown in Fig. 4. UCAM-70 software was used for data collection and live monitoring of the load displacement relationship.

### Test procedure

**Figure 5:**
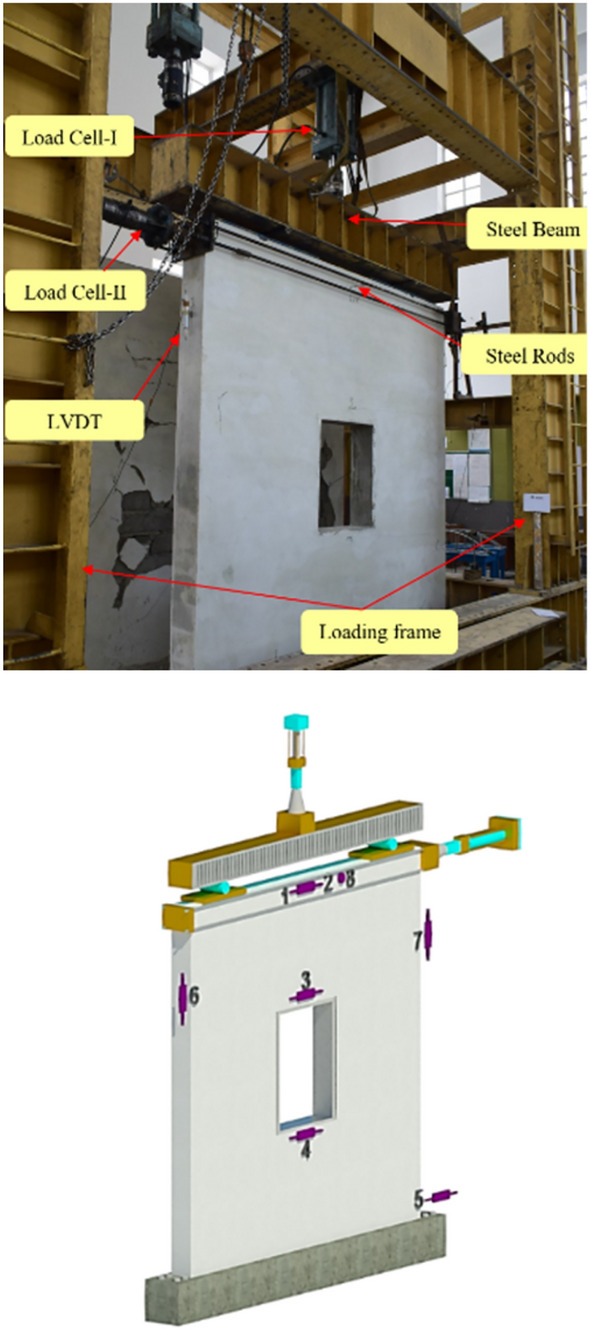
Test setup.

**Figure 6:**
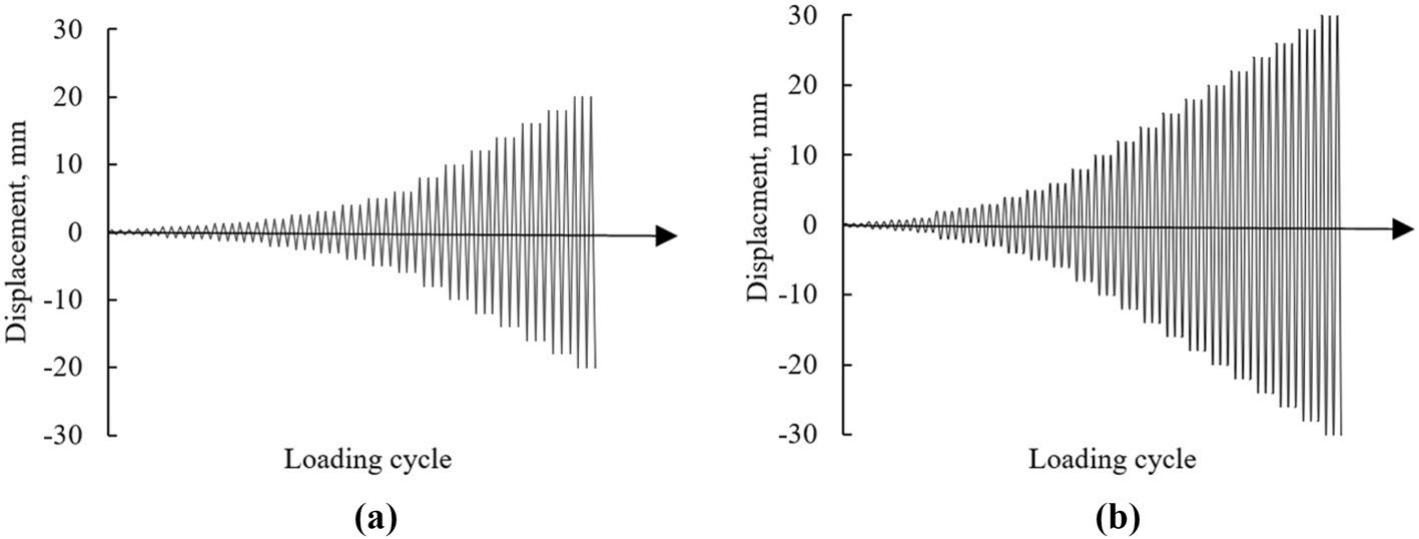
Displacement controlled testing (**a**) URM (**b**) CM.

Tests were performed in displacement control environment and different displacement increments, listed in Table 3, were applied as per the criteria of FEMA-461^39^ and already used by different researchers^40–42^. Displacements were applied in increments and corresponding load was recorded through load cell. QSRCL test was performed on both specimens to evaluate the seismic resistant behavior and parameters. QSRCL tests were used many times by researchers to evaluate the seismic performance of low compressive strength masonry units^1,2^. QSRCL test setup of both specimens are shown in Fig. 5. Walls were subjected to the combination of constant vertical (pre-compression) load and a reverse cyclic lateral load. Three displacement cycles were applied to get a more stable damaged state at each storey drift, and cracks were marked and carefully observed at the end of each cycle. Typically applied displacement on specimens is shown in Fig. 6. The lateral load was applied at the top beam, where a steel box along with four steel rods were placed to fasten it with bolts, to bring the wall to its original position during pull. The tests were continued until 20% strength degradation occurred, or the specimen get extremely damaged, whichever happened first^39^.

**Table 3:** Displacements applied on the walls.

Steps of displacement	Cycles	Cumulative no. of cycles	Displacement mm (% storey drift) of URM	Displacement mm (% storey drift) of CM
1	3	3	0.25 (0.01)	0.25 (0.01)
2	3	6	0.5 (0.02)	0.5 (0.02)
3	3	9	0.75 (0.02)	0.75 (0.02)
4	3	12	1 (0.03)	1 (0.03)
5	3	15	1.25 (0.04)	1.25 (0.04)
6	3	18	1.5 (0.05)	1.5 (0.05)
7	3	21	2 (0.07)	2 (0.07)
8	3	24	2.5 (0.08)	2.5 (0.08)
9	3	27	3 (0.10)	3 (0.10)
10	3	30	4 (0.13)	4 (0.13)
11	3	33	5 (0.16)	5 (0.16)
12	3	36	6 (0.20)	6 (0.20)
13	3	39	8 (0.26)	8 (0.26)
14	3	42	10 (0.33)	10 (0.33)
15	3	45	14 (0.46)	14 (0.46)
16	3	48	16 (0.52)	16 (0.52)
17	3	51	18 (0.59)	18 (0.59)
18	3	54	–	20 (0.66)
19	3	57	–	22 (0.72)
20	3	60	–	24 (0.79)

## Results and discussion

The results of both specimens are discussed according to QSRCL's loading scheme and testing protocols. Data from various gauges is analyzed to evaluate seismic behavior and achieve target parameters. The paragraph covers damage patterns, failure mechanisms, and hysteric behavior, including bi-linear idealization and performance levels at different damage states. The specimens' lateral strength, stiffness degradation, energy dissipation, ductility, and damping are assessed. Gauges measuring in-plane sliding, out-of-plane displacement fields, and rocking are also discussed. Finally, a comparison between both walls' seismic behavior and resistance parameters is made.

### Damages in CM wall

To achieve a stable damaged pattern, each specimen underwent three cycles of displacement for every storey drift. At the end of the third cycle, cracks were marked while the specimens were held at maximum displacement. Results showed that CM and URM walls had linear elastic behavior up to a maximum storey drift of 0.07% and 0.05%, respectively. The diagonal shear cracks in CM walls originated from the corner of the opening at a storey drift of 0.07%. Plaster spalling occurred at a storey drift of 0.19%. Cracks density increased below the lintel beam at a storey drift of 0.46%. Piers suffered significant damage, while spandrels had limited cracks parallel to horizontal bed joints due to low pre-compression load and weak bond between mortar and masonry units. Confining elements were not affected by cracks as weak masonry units were already extensively damaged. The test was stopped when strength degradation of around 20% (62.42 kN) was reached at a storey drift of 0.79%.

### Damages in URM wall

The URM wall was tested with the same loading protocols and boundary conditions. Due to low pre-compression load, a crack appeared at the bottom course indicating weak bond between mortar and masonry unit. Rocking failure occurred at 0.05% storey drift, causing damage to the opposite side's toe of the wall. The crack gradually propagated towards the other corner of the wall, and failure behavior shifted from flexural to shear as displacement increased. Shear sliding occurred at the horizontal bed joint, which is a stable mechanism. It is also witnessed in the past, that rocking and shear sliding frequently take place together in the presence of low pre-compression load^43^ and poor quality of mortar^44^. The wall still resisted lateral loading, but at a decreasing rate. The test was stopped at 0.60% storey drift, when the wall's lateral load capacity reduced to 80% of its peak load and global integrity was disturbed.

The confining elements enhanced the cracks density in the CM wall, which hold the masonry units, and led to the gradual and steady occurrence of cracks^45^. On the other hand, cracks in the URM wall happened sequentially such as rocking followed by shear sliding and diagonal shear. The overall failure modes were the same for both specimens, but the sequence of occurrence was different from each other. In general, shear failure was found to be dominant in both specimens. Various storey drift ratios and corresponding loads with damaged description has been summarized in Table 4.

**Table 4:** Summarized damage patterns of CM and URM wall.

Storey drift ratio (%)	Load (kN)	Damage description in failure modes
Confined masonry wall		
0.07	42.23	Diagonal shear cracks were originated from the corner of the wall, but which were very small as shown Fig. 7a
0.09	63.83	Up to this storey drift ratio the behavior was linear elastic and diagonal shear crack was occurred as depicted in Fig. 7b
0.19	78.00	Diagonal shear cracks were propagated along with plaster dispatching as show in Fig. 7c
0.26	77.76	Same diagonal shear cracks as before, and no cracks in spandrel level till this storey drift ratio as shown in Fig. 7d
0.46	71.56	Diagonal shear cracks were further propagated, and density of cracks were increased as depicted in Fig. 7e
0.79	62.41	Diagonal shear cracks were propagated further while the density of cracks increased, although there were no cracks in confining elements. The most severe cracks were occurred in the piers as depicted in Fig. 7f
Unreinforced masonry wall		
0.05	34.56	Overall, the behavior of the wall remained linear elastic. Due to weak bond between mortar and masonry units shear sliding were started, but not extended to significantly distance as shown in Fig. 8a
0.06	36.87	Rocking failure was started at this level due to low pre-compression load and weak bond between mortar and masonry units as shown in Fig. 8b
0.52	38.32	Diagonal shear failure occurred at this storey drift ratio as shown in Fig. 8c
0.60	29.98	Rocking, shear sliding, and diagonal shear were the dominated failure modes at this stage; however, the integrity of the wall was disturbed as depicted in Fig. 8d

The seismic capacity CLCBM can be compared with conventional brick masonry, tested at the University of Engineering and Technology, Peshawar^40^. The overall configuration of these walls is not identical as that of CLCBM walls. The force–deformation parameters have been given in Table 5. Significant differences have been observed in the overall performance and capacity of these walls. CLCBM walls show high post-crack ductility due to CLC blocks. However, the maximum capacity of the CLCBM walls is comparatively lower than conventional brick masonry walls as given in Table 5. Post-crack stiffness degradation of CLCBM walls is very rapid due to weak masonry units. In addition, the elastic stiffness of CLCBM walls is very good and comparable with conventional brick masonry walls. The post-crack ductility of conventional brick masonry walls is lesser as compared to the remaining two walls made of CLC blocks. However, the maximum capacity of the brick masonry walls is higher than CLCBM walls.

**Table 5:** Parametric comparison of different CM and URM walls.

Parameters	Confined masonry walls		Unreinforced masonry Walls		Ratio
	CLC block	Conventional brick ^37^	CLC block	Conventional brick ^37^	(CLC CM/CLC URM)
Peak load (kN)	78.02	93	38.86	39.6	2.00
Displacement ductility ratio, µ_d_	15.80	–	15.00	–	2.02
Yield strength (kN)	70.22	83.7	34.78	35.64	1.25
Yield displacement, ${\Delta }_{y}$ (mm)	1.52	2.85	1.22	2.61	1.56
Ultimate displacement, ${\Delta }_{u}$ (mm)	24	17.06	18	9.75	1.05
Elastic stiffness (kN/mm)	47.25	33	28.35	15.14	1.67

**Figure 7:**
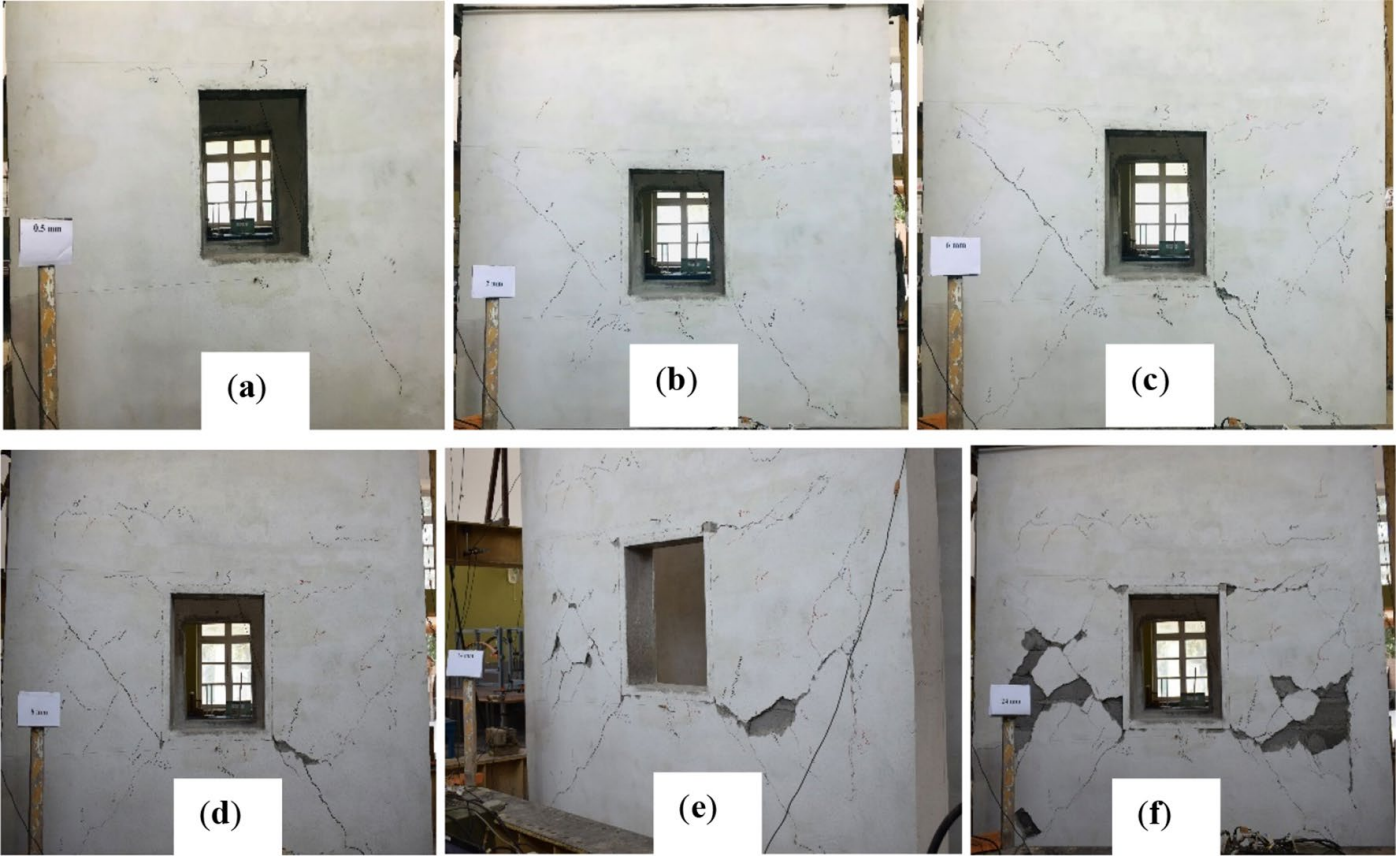
Damaged state of CM wall at storey drift ratios of (**a**) 0.02, (**b**) 0.06, (**c**) 0.19, (**d**) 0.26, (**e**) 0.46, and (**f**) 0.79.

**Figure 8:**
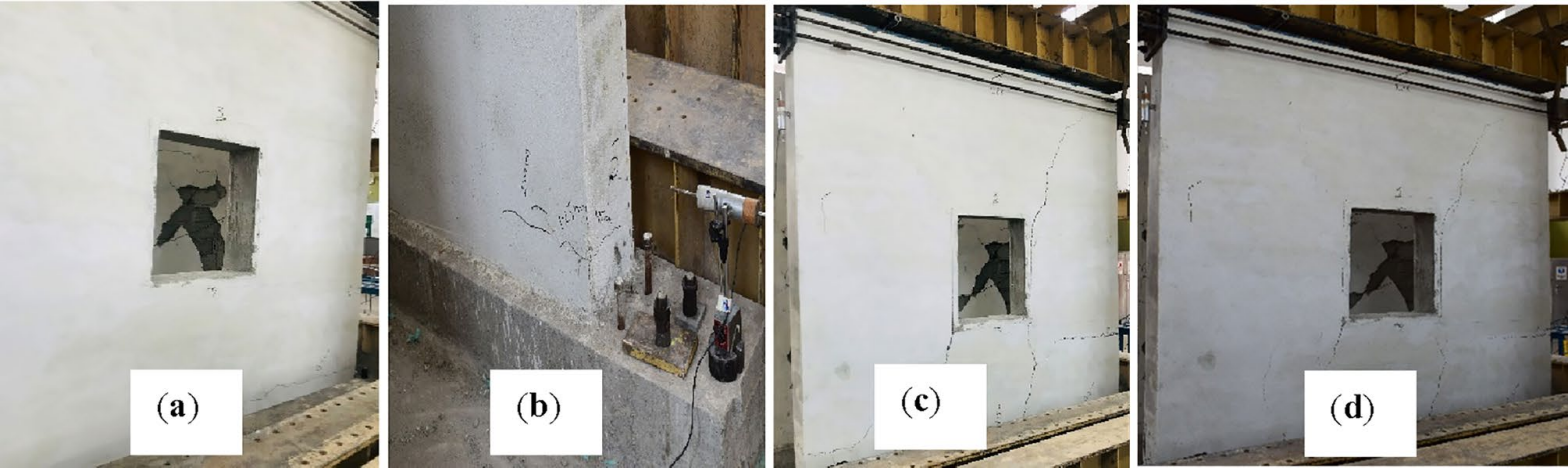
Damaged state of URM wall at storey drift ratios of (**a**) 0.04, (**b**) 0.06, (**c**) 0.52 and (**d**) 0.60.

**Figure 9:**
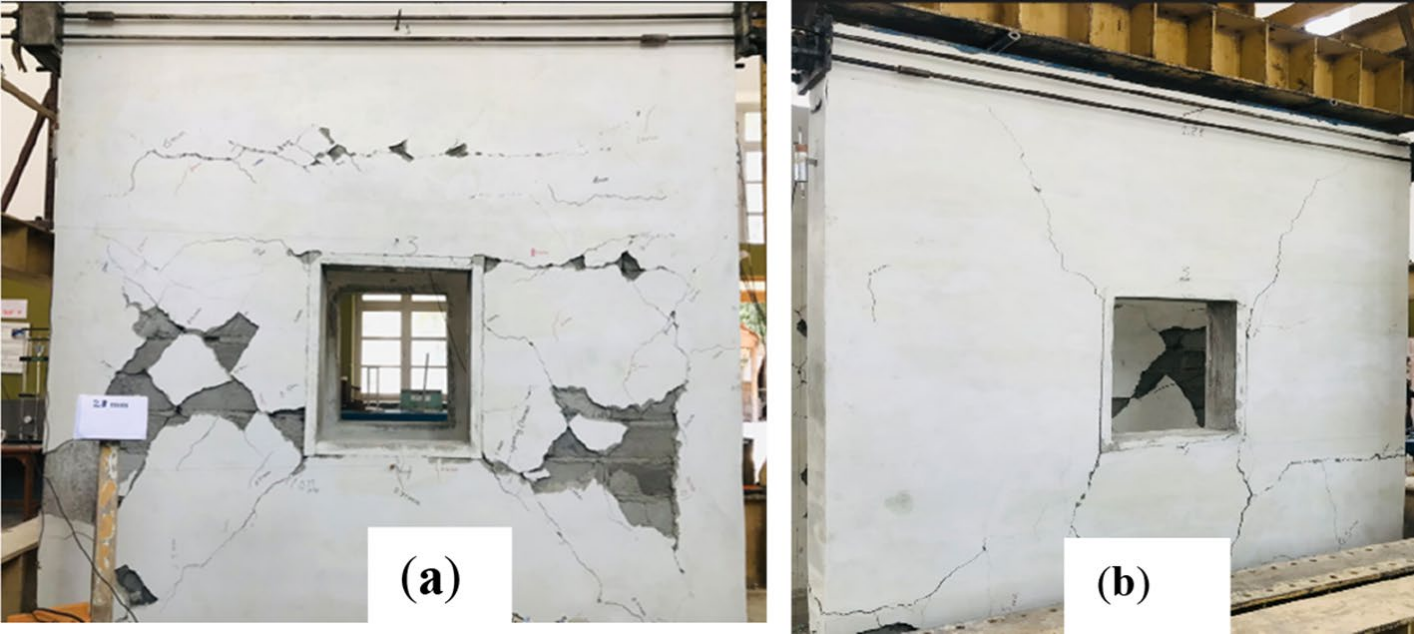
Final damaged state of (**a**) CM wall and (**b**) URM wall.

The final crack patterns of both specimens are depicted in Fig. 9.

### Force–deformation behavior and hysteresis loops

**Figure 10:**
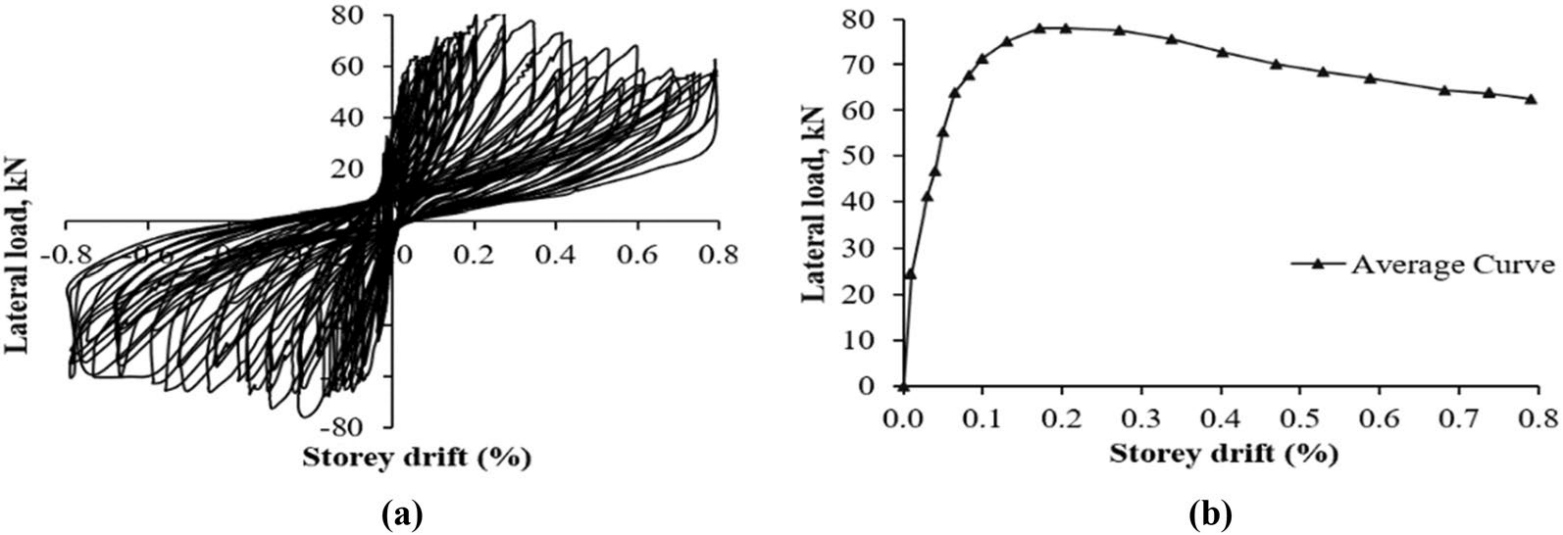
Response of CM wall (**a**) hysteresis loops; (**b**) force–deformation curve.

**Figure 11:**
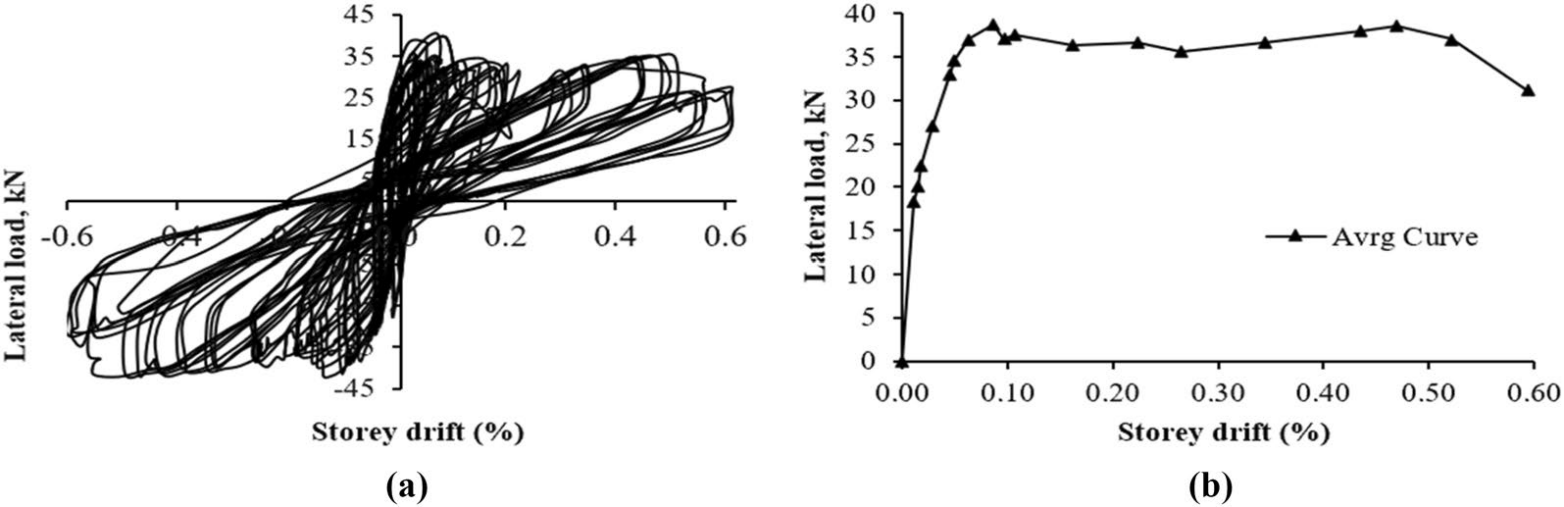
Response of URM wall (**a**) hysteresis loops; (**b**) force–deformation curve.

Hysteresis loops and envelope curves were created for both specimens using lateral load and storey drift measurements. The load was measured with a horizontal load cell, while displacement was averaged from LVDTs 1 and 2. The envelope curve was formed by connecting peak resistive loads and corresponding storey drift ratios. The force–deformation hysteresis behavior is shown in Figs. 10 and 11 and discussed further below.

#### CM wall

Hysteresis loops at lower storey drift ratios are tight until 0.08%, indicating low energy dissipation as shown in Fig. 10. Despite damage, the specimen resisted up to 0.20% drift and 78.02 kN load. Beyond this point, wider loops showed high energy dissipation and stiffness degradation. Weak masonry units and interface bond led to this degradation. Wall failure was due to diagonal shear, followed by rocking and shear sliding.

#### URM Wall

Figure 11 shows the hysteresis loops of URM wall. The storey drift ratios plotted are based on horizontal maximum displacement at the top without correction for shear sliding. Tight loops were observed initially until a storey drift of 0.04%, indicating less energy dissipation due to fewer cracks. Rocking and shear sliding occurred at the bottom of the wall due to low pre-compression load and weak interface bond. Pure rocking can lead to high deformation capacity^43,46^. The wall exhibited elasto-perfect-plastic behavior due to rocking and shear sliding followed by X-type or diagonal shear cracks. Post-peak response showed high ductility and constant stiffness, but the stiffness and force resistance abruptly fell at the end due to sudden diagonal shear cracks. This behavior is difficult to control and rare in practical conditions^43^. Later cycles showed widened loops due to more cracks and energy dissipation.

### Bi-linear idealization

**Figure 12:**
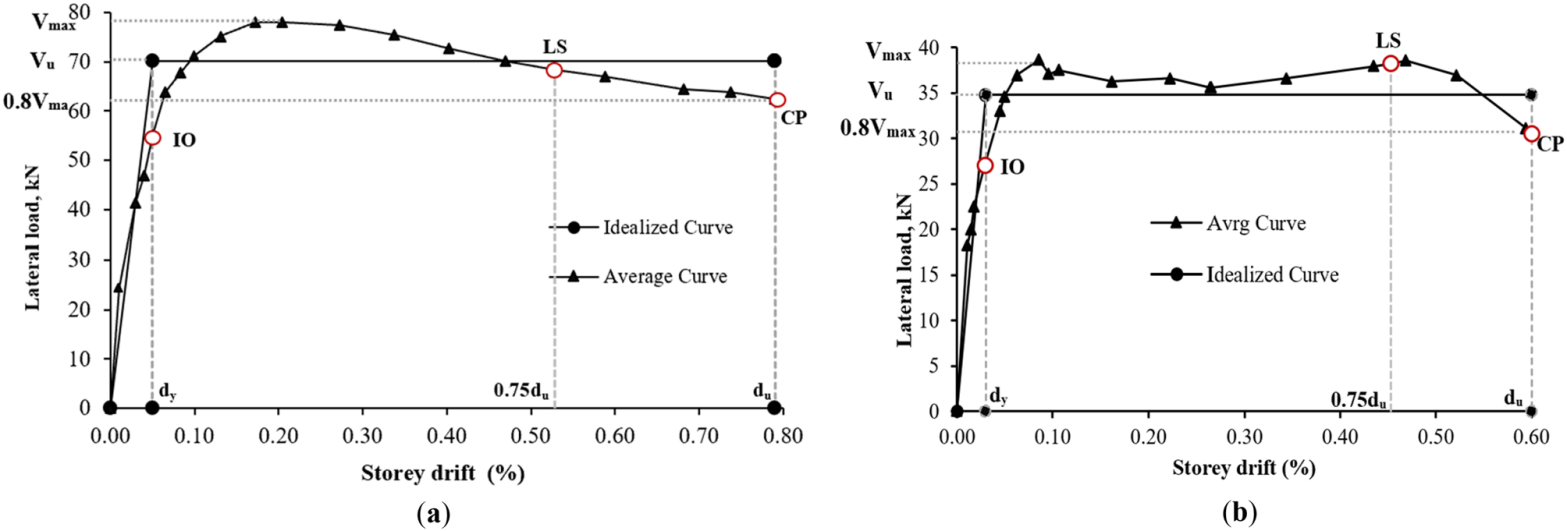
Force–deformation and bilinear idealized curve of (**a**) CM wall and (**b**) URM wall.

The study simplified force deformation curves using bi-linear idealization based on the equal energy principle. Both bi-linear and tri-linear idealization was recommended by Tomazovic^33^ and Magenes and Calvi^43^. Bi-linear idealization was chosen for simplicity, and crucial points such as effective stiffness (*K*_*e*_), ultimate displacement capacity (*d*_*u*_), and shear load (*V*_*u*_) were identified. *K*_*e*_ values were 47.25 kN/mm for CM and 28.35 kN/mm for URM walls. The *d*_*u*_ corresponds to a 20% strength degradation, and the *V*_*max*_/*V*_*u*_ ratio is 0.9, which is in close agreement with the range of 0.90–0.95 proposed by Tomazovic^33,47^. Figure 12 shows the average force–deformation envelope and bi-linear idealized curve for both specimens.

1 $${K}_{e}=\frac{{0.75V}_{u}}{{d}_{0.75{V}_{u}}}$$

where ${d}_{0.75{V}_{u}}$ is displacement corresponding to 0.75*V*_*u*_. For CM and URM walls, the corresponding values of ultimate and yield storey drift are 0.79%, 0.05%, and 0.60%, 0.04%, respectively. Displacement ductility was calculated using Eq. (2).

2 $${\mu }_{d}=\frac{{d}_{u}}{{d}_{y}}$$

The ${\mu }_{d}$ value for CM and URM walls are 15.80 and 15.00, respectively. The reason for such high values of displacement ductility is attributed to diagonal shear failure and rocking flexure failure in both specimens^48^. There are several approaches available in the literature for the determination of response modification factor (R) or generally used term are force reduction factor (by European standard structural behavior factor, q)^49–52^. However, for simplicity, the method widely used and proposed by Newmark et al.^53,54^, was considered for this study. As per the stated approach, R can be calculated using Eq. (3). R values are 5.53 and 5.38 for CM and URM walls, respectively.

3 $$R= \sqrt{2\mu -1}$$

Different values of structural behavior proposed in Eurocode 8^55^ for different conventional masonry structures such as for unreinforced masonry (R = 1.5–2.5), confined masonry (R = 2.0–3.0) and reinforced masonry (R = 2.5–3.0)^52^. However, in this study, some higher values were found, which was due to weak masonry units and types of failure, that occurred in the specimens, due to which the ultimate displacement was increased significantly. The parametric comparison of both specimens is given in Table 5.

### Structural performance levels

**Figure 13:**
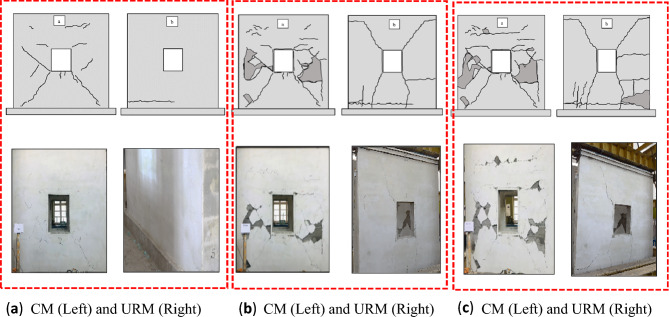
Cracks pattern at (**a**) IO level, (**b**) LS level and (**c**) CP level.

Figure 13 depicts the cracks propagation at different performance levels. The performance levels include immediate occupancy (IO), life safety (LS), and collapse prevention (CP) are determined as per the ASCE 41-02^56^. Three performance levels IO, LS, and CP have been identified and marked on the force–deformation curves for both specimens. The performance levels, corresponding to different storey drifts, loads, and damages details have been given in Table 6.

**Table 6:** Performance levels and damages.

Performance level	Storey drift ratio (%)	Load (kN)	Damage description and failure modes
Confined masonry wall			
IO	0.05	55.26	Cracks were originated from the corner of the opening, which showed the diagonal shear mode
LS	0.52	68.23	Cracks density were increased in piers, while dispatching of plaster was also noted
CP	0.79	62.22	Significant cracks were observed in piers along with some sliding noted in spandrel
Unreinforced masonry wall			
IO	0.04	27.14	A crack occurred at the bottom course, which showed rocking behavior
LS	0.45	38.23	Toe crushed and horizontal cracks were extended and finally transformed to sliding
CP	0.60	31.00	At CP, 20% capacity degradation was achieved. In the end, a diagonal shear crack was occurred

### Energy dissipation mechanism

**Figure 14:**
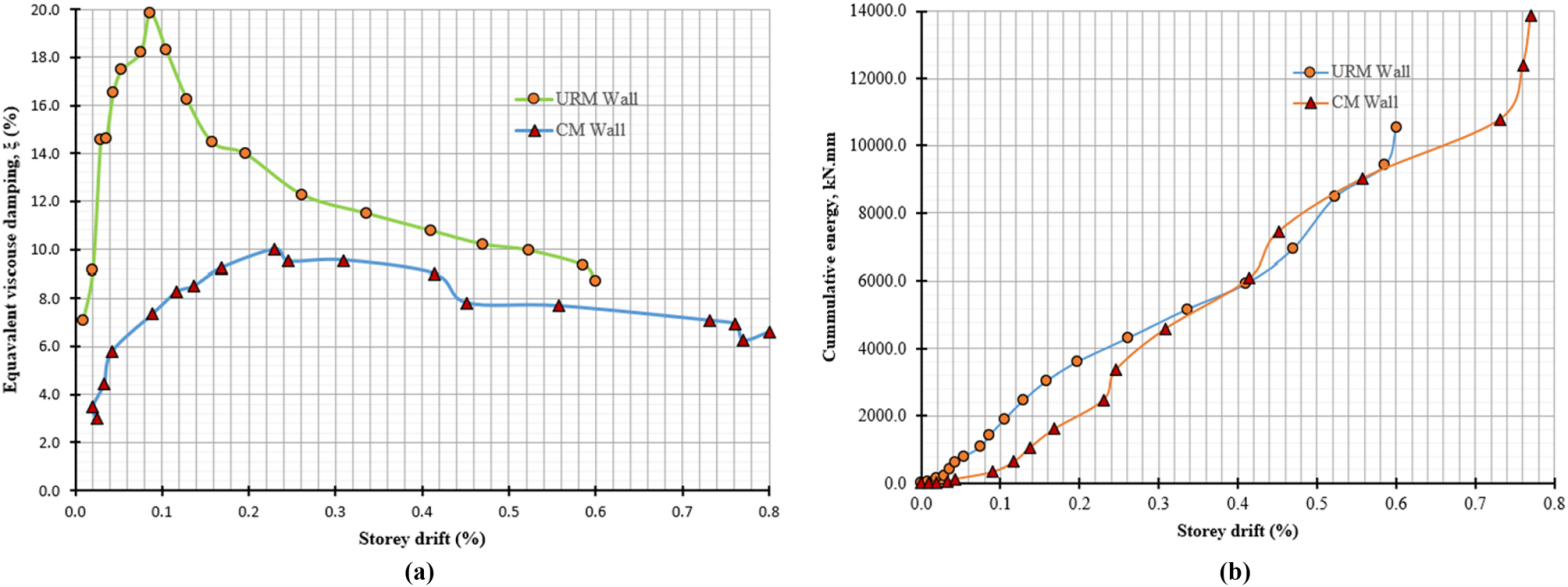
(**a**) Equivalent viscous damping vs storey drift (**b**) cumulative energy vs storey drift.

Figure 14a shows the damping behavior of both walls. The dissipated hysteretic energy of specimens was scrutinized in terms of equivalent viscous damping, ${\xi }_{eq}$. The equivalent viscous damping coefficient was calculated using the formulation proposed by Anil Ka Chopra^51^, as given in Eq. (4). Equivalent viscous damping characterized the amount of energy dissipated during the testing. Equivalent viscous damping shows the relationship between energy dissipated per cycle to the input energy put into the structure to achieve the target-imposed displacement on the structure. Input energy is the amount of energy needed for structure to achieve target displacement and employs in the form of strain energy

4 $${\xi }_{eq }= \frac{{E}_{d}}{{2\pi E}_{inp}}$$

where *E*_*d*_, dissipated energy per cycle (which is the average area enclosed by hysteresis loops of three cycles per storey drift ratio) and *E*_*inp*_, is the amount of energy required to be embedded in structure to achieve target displacement, it can be calculated as half product of the peak load and corresponding displacement at each cycle.

Initially, the specimens show high stiffness and with increasing energy dissipation. The shear failure dissipates more energy, due to which the graph shows an increasing trend, while in later cycles, the magnitude of imposed displacement was increased, which compressed the weak masonry units and did not allow to dissipate much energy. Therefore, a decreasing trend of equivalent viscous damping of both samples was observed. In later cycles, when the contribution of interface friction and strength of masonry units ended, the graph was almost straightened in CM wall, which is not happing in conventional masonry units. Therefore, the energy dissipation of these masonry units is less.

Figure 14b shows that initially in elastic limit, the energy accumulated in URM wall is more as compared to CM wall. However, in later cycles, the damaged pattern and behavior of both specimens were different from each other, therefore, the pattern of the cumulative energy in both specimens are different. Overall, the energy accumulated in CM is more than that of URM wall due to confining elements.

### Stiffness degradation

**Figure 15:**
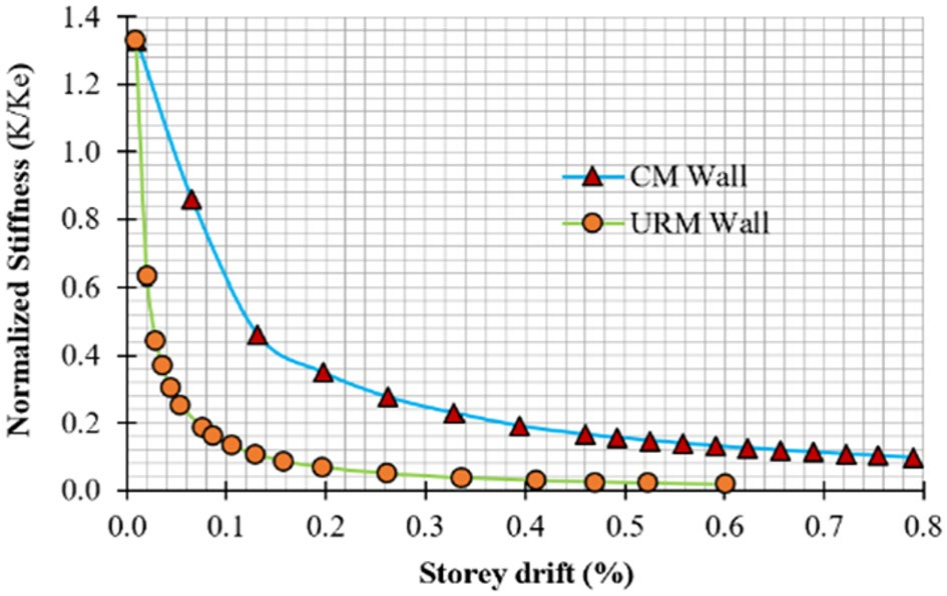
Normalized stiffness vs storey drift.

Stiffness degradation is caused by cracks and structural damage during displacement. To measure stiffness degradation in masonry structures, normalized stiffness (*K/K*_*e*_) is plotted on the y-axis and storey drift on the x-axis. Normalized stiffness is the ratio of average stiffness (*K*) to effective or elastic stiffness (*K*_*e*_), obtained from a bi-linear idealized curve against the damaged index^57^ or storey drift^58^. Figure 15 shows the overall stiffness degradation behavior of both specimens under lateral load. Stiffness degradation is higher at lower storey drift ratios due to crack formation. Although degradation continues at a lower rate in subsequent cycles, the pace slows. The stiffness degradation rate rapidly decreases at a storey drift of 0.1% and 0.2% for URM and CM walls, respectively. Beyond this limit, degradation occurs at a low rate until it becomes negligible at the end of the test. Cracks cause disturbance in the global rigidity of the specimens and are responsible for the high rate of stiffness degradation. At 0.13% storey drift, CM and URM walls experienced 65.41% and 91.72% stiffness degradation, respectively. URM walls experience a higher rate of stiffness degradation due to the lack of confining elements^59^. At the end of the test, CM walls experienced 92.48% degradation, while URM walls experienced 98.49% degradation.

### Global rocking, In-plane sliding, and out-of-plane movement of the walls

**Figure 16:**
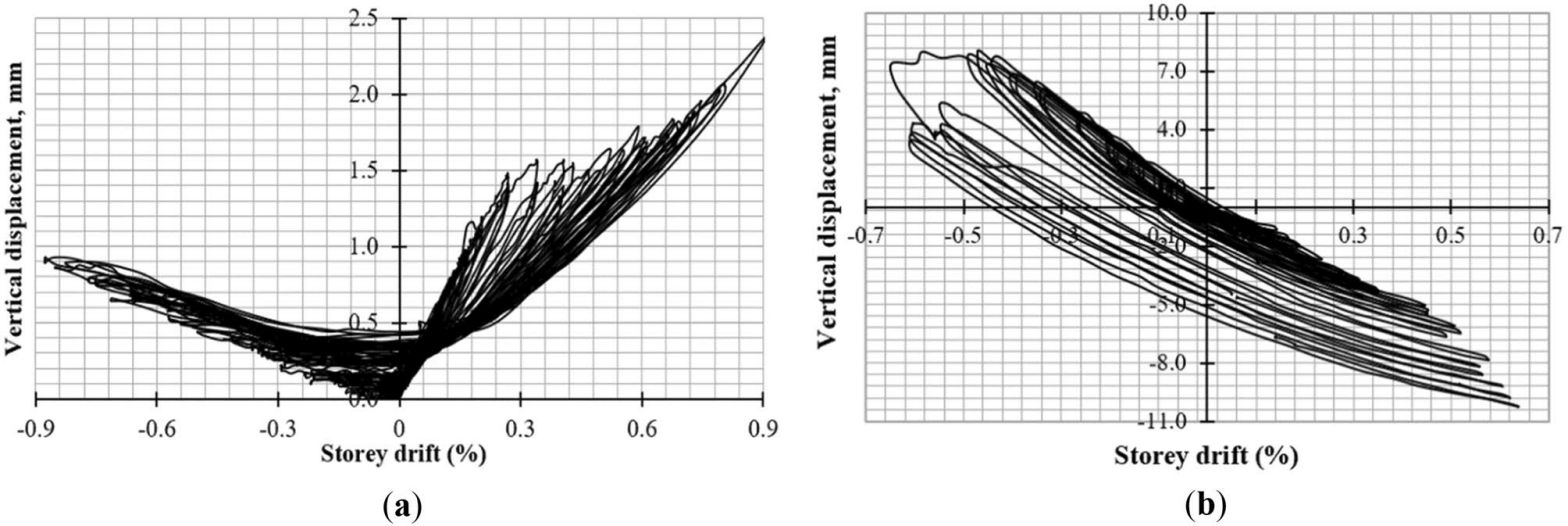
Rocking behavior of (**a**) CM wall; (**b**) URM wall.

To monitor global rocking, two LVDTs (6 and 7) were installed at each end of the walls. The CM wall had minimal rocking behavior as depicted in Fig. 16a, but unsymmetrical behavior resulted in more cracks in positive cycles. The URM wall exhibited symmetrical behavior with significant rocking due to toe failure in compression and shear sliding caused by low interface friction between masonry units and mortar as shown in Fig. 16b.

**Figure 17:**
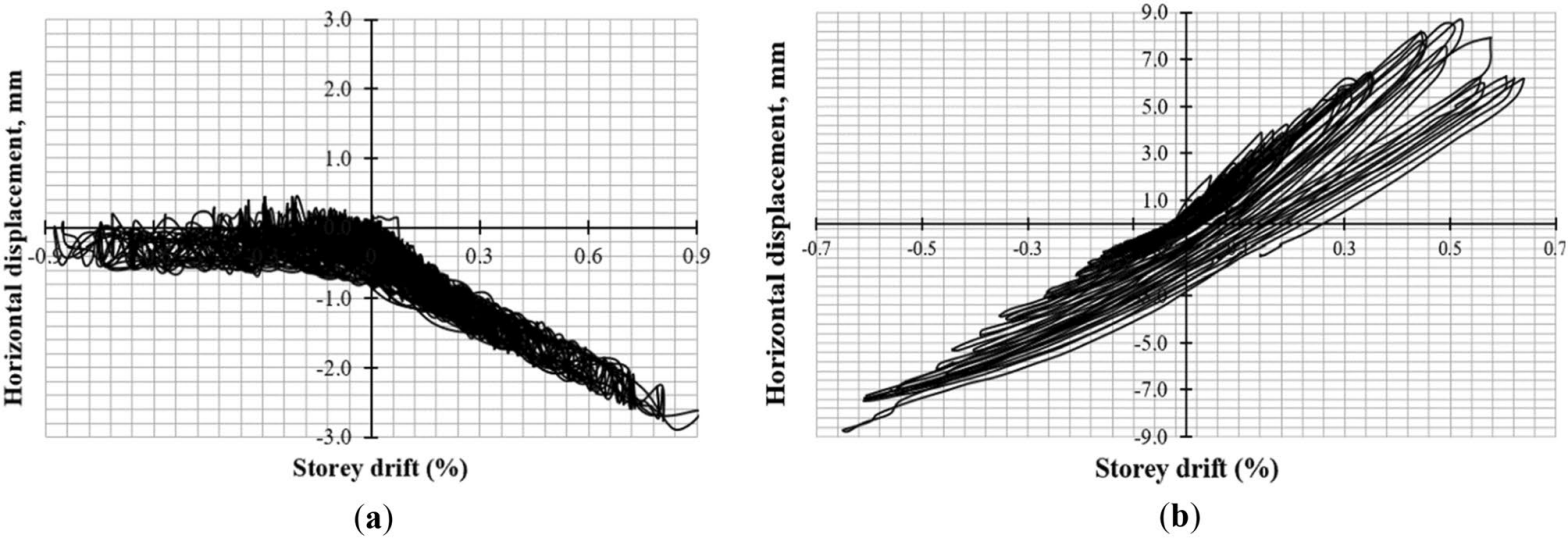
In-plane sliding of (**a**) CM wall; (**b**) URM wall.

In-plane sliding displacement was monitored through LVDT-5, with the CM wall exhibiting unsymmetrical behavior due to non-uniform cracks in the north and south piers as shown in Fig. 17a. The URM wall showed significant in-plane sliding due to shear sliding and rocking failure mode as depicted in Fig. 17b.

**Figure 18:**
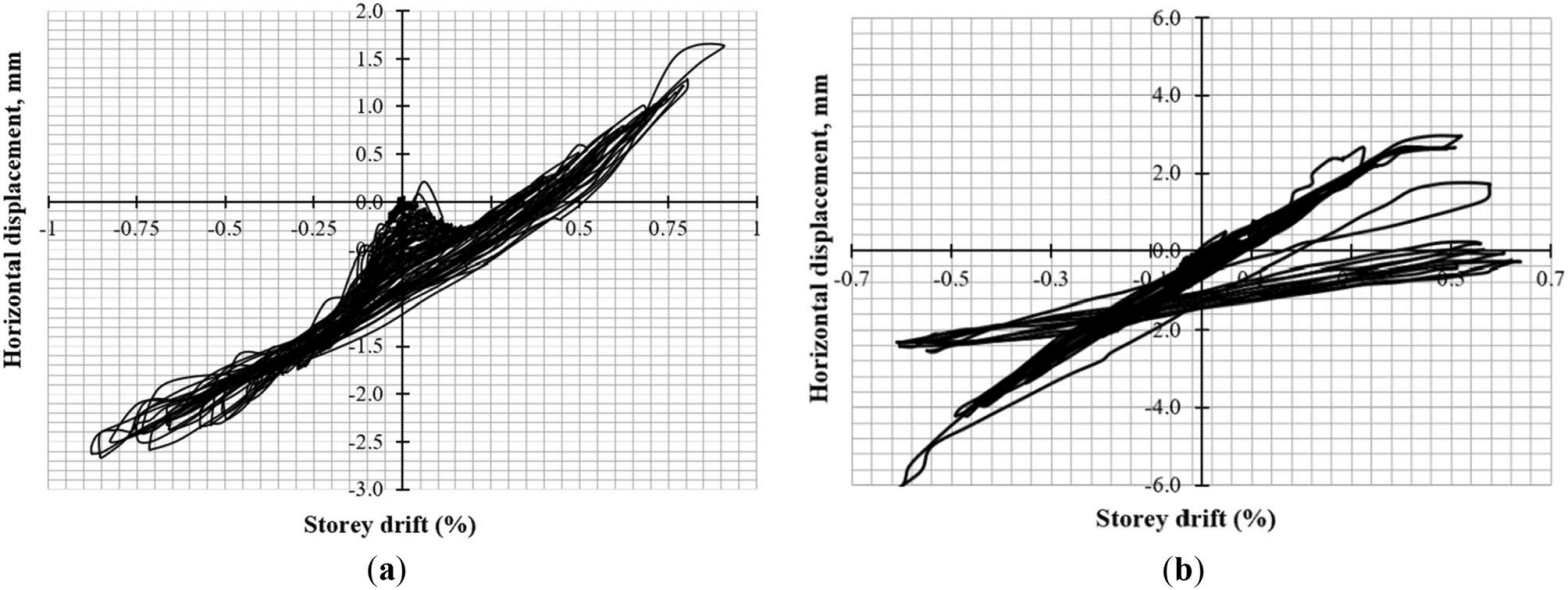
Out-of-plane displacement of (**a**) CM wall; (**b**) URM wall.

Out-of-plane movement was monitored through LVDT-8, with negligible out-of-plane displacement occurring in the CM wall due to confining elements^60^ as shown in Fig. 18a. Symmetrical behavior was observed in the URM wall with maximum out-of-plane displacement of 6.0 mm occurring at a storey drift of 0.60% due to in-plane sliding followed by sudden diagonal shear failure as depicted in Fig. 18b.

## Conclusions

The objective of this study has to evaluate the seismic performance of confined and unreinforced CLCBM walls. QSRCL test was performed on each specimen and data was analyzed to evaluate the seismic performance in terms of hysteresis loops, force–deformation, energy dissipation, stiffness degradation, response modification factor R, and deformation ductility factors *μ*_*d*_. The sequence and mechanism of damages were also observed during testing. After careful data analysis and results interpretation, the following conclusions are drawn.

- The specimens failed in hybrid mode, with shear failure mode being considered as dominant. Although the sequence of occurrence of various failure modes are different. The CM wall failed in diagonal shear, shear sliding and rocking faire mode, while the URM wall failed in rocking, shear sliding, and diagonal shear mode.
- The CM wall system was found to be effective in significantly improving the seismic performance of the URM wall, by enhancing its lateral load capacity.
- The seismic performance and capacity of the CM wall are significantly higher than the URM wall. The lateral load capacity, elastic stiffness, and displacement ductility of the CM wall were increased by 102%, 66.67%, and 5.3%, respectively as compared to URM wall.
- Considerable impact of confining elements was observed on energy dissipation and stiffness degradation but in later storey drifts it reduces.
- Due to weak masonry units no evident difference was observed in the ductility of both wall specimens.
- Confining elements contribute greatly to the deformation capacity of CM as compared to URM wall.

Research work can be carried out on the performance evaluation of CLCBM walls in out-of-plane direction. In addition, numeral modeling can be carried out to validate the results.

## Data Availability

The datasets used and/or analyzed during the current study available from the corresponding author on reasonable request.
